# Outcome of patients with tetralogy of Fallot with pulmonary atresia

**DOI:** 10.3389/fped.2022.1077863

**Published:** 2023-01-30

**Authors:** Eva Sames-Dolzer, Anna Fahrnberger, Michaela Kreuzer, Roland Mair, Gregor Gierlinger, Andreas Tulzer, Roland Gitter, Christoph Prandstetter, Gerald Tulzer, Rudolf Mair

**Affiliations:** ^1^ Division of Pediatric and Congenital Heart Surgery, Kepler University Hospital, Linz, Austria; ^2^ Medical Faculty, Johannes Kepler University Linz, Linz, Austria; ^3^ Department of Pediatric Cardiology, Kepler University Hospital, Linz, Austria

**Keywords:** tetralogy of Fallot, pulmonary atresia, aorto-pulmonary collaterals, unifocalization, rehabilitation, staged repair

## Abstract

**Objective:**

Tetralogy of Fallot patients with pulmonary atresia (TOFPA) have a largely varying source of pulmonary perfusion with often hypoplastic and even absent central pulmonary arteries. A retrospective single center study was undertaken to assess outcome of these patients regarding type of surgical procedures, long-term mortality, achievement of VSD closure and analysis of postoperative interventions.

**Methods:**

76 consecutive patients with TOFPA operated between 01.01.2003 and 31.12.2019 are included in this single center study. Patients with ductus dependent pulmonary circulation underwent primary single stage full correction including VSD closure and right ventricular to pulmonary conduit implantation (RVPAC) or transanular patch reconstruction. Children with hypoplastic pulmonary arteries and MAPCAs without double supply were predominantly treated by unifocalization and RVPAC implantation. The follow up period ranges between 0 and 16,5 years.

**Results:**

31 patients (41%) underwent single stage full correction at a median age of 12 days, 15 patients could be treated by a transanular patch. 30 days mortality rate in this group was 6%. In the remaining 45 patients the VSD could not be closed during their first surgery which was performed at a median age of 89 days. A VSD closure was achieved later in 64% of these patients after median 178 days. 30 days mortality rate after the first surgery was 13% in this group. The estimated 10-year-survival rate after the first surgery is 80,5% ± 4,7% showing no significant difference between the groups with and without MAPCAs (*p* > 0,999). Median intervention-free interval (surgery and transcatheter intervention) after VSD closure was 1,7 ± 0,5 years [95% CI: 0,7–2,8 years].

**Conclusions:**

A VSD closure could be achieved in 79% of the total cohort. In patients without MAPCAs this was possible at a significant earlier age (*p* < 0,01). Although patients without MAPCAs predominantly underwent single stage full correction at newborn age, the overall mortality rate and the interval until reintervention after VSD closure did not show significant differences between the two groups with and without MAPCAs. The high rate of proven genetic abnormalities (40%) with non-cardiac malformations did also pay its tribute to impaired life expectancy.

## Introduction

Patients with tetralogy of Fallot and pulmonary atresia (TOFPA) lack an antegrade flow *via* the right ventricular outflow tract. The total cardiac output is ejected *via* the enlarged aortic valve and the pulmonary blood flow derives from several different spots of the aorta. The pulmonary vascular bed shows a wide range of variety (1). At the milder end of the spectrum, a subgroup of this population has an open ductus arteriosus which supplies mostly well-developed central pulmonary arteries. On the other end, the central pulmonary arteries may be diminutive or may be missing completely and the pulmonary perfusion is established *via* major aorto-pulmonary collateral arteries (MAPCAs) originating most frequently from the descending aorta. This variety requires an individualized surgical approach targeting a satisfying development of the pulmonary artery tree and the closure of the ventricular septal defect (VSD).

In case of adequate size of the pulmonary arteries, the VSD closure can be performed at neonatal age as part of a single stage full correction repair with establishment of a right ventricular to pulmonary artery (RV-PA) connection. The early procedure leads to fast normalization of the circulation with lesser organ impairment, but neonatal on pump surgery might carry a higher perioperative risk and implies a smaller conduit size. Subsequent catheter interventions and reoperations at the level of the RV-PA conduit or the pulmonary arteries are common.

Patients with hypoplastic pulmonary arteries need increased pulmonary blood flow to stimulate growth of the vasculature in order to enable a VSD closure at a later date. Different surgical approaches have been reported which either pursue a unifocalization strategy of collaterals as suggested by the Stanford (2) and Birmingham teams (3, 4) or a rehabilitation strategy of the hypoplastic native pulmonary arteries as promoted by the Melbourne group (5, 6). A combined strategy tries to unite both pathways (7) and several studies include different management protocols as individualized procedures dependent on the underlying anatomy.

At our center these patients undergo a staged repair aiming at primary creation of a pulmonary vascular tree as big as possible with inclusion of all necessary MAPCAs and connection to the right ventricular outflow tract using a valved conduit. As an alternative, shunt procedures to the central pulmonary arteries were performed as a primary palliation in extreme cases with diminutive native pulmonary arteries. After growth of the pulmonary bed the pulmonary resistance might be low enough to make the VSD closure possible with an acceptable risk. Due to recurrent severe stenoses of the pulmonary arteries or regions with pulmonary hypertension some patients never reach this status and have to continue living with cyanosis caused by a right to left shunt of increasing degree. In these patients, surgical palliations can be performed to help improve their general condition and quality of life.

After unifocalization of MAPCAs catheter interventions are often inevitable to treat the hypoplastic vessels and analyze the pulmonary resistance. In order to objectify the probability of a successful VSD closure a standardized intraoperative measurement protocol of the pulmonary artery pressure is recommended (8–10) and was also implemented recently at our center.

This retrospective single center study of our total TOFPA cohort was undertaken to investigate and compare the outcome of patients with and without MAPCAs. Data were collected regarding type and timing of surgical procedures, long term mortality, achievement of VSD closure and analysis of postoperative interventions.

## Methods and patients

In this investigation data were collected of all consecutive patients with tetralogy of Fallot and pulmonary atresia who were operated between 1.1.2003 and 31.12.2019 at the Children's Heart Center Linz. In total 76 patients could be included into the study, 52,6% of them were male. 44,3% of the babies were diagnosed prenatally and referred to our center during pregnancy.

In 40% of the patients a genetic malformation could be proven, a 22q11 deletion was diagnosed in 25% of the children. The main associated malformations of the great vessels were a right aortic arch in 36%, an aberrant right subclavian artery in 14% and coarctation/hypoplastic aortic arch or aortic valve dysplasia in 4%.

The cardiac diagnosis was made using echocardiography and in cases with suspicion of MAPCAs an early cardiac catheterization was performed in order to illustrate number, size, origin and distribution of the MAPCAs and depiction of the native central pulmonary arteries. In presence of a ductus arteriosus and good clinical status an early surgery was performed aiming at complete correction depending on vessel anatomy. Pulmonary artery diameter of at least 3 mm on either side at neonatal age and absence of MAPCAs should allow single stage full correction in PDA children under stable clinical conditions. As an alternative, ductal stenting can be considered in high risk PDA children. In patients with MAPCAs and stable condition surgery was postponed to a later date (at least 6 weeks, but usually several months) to minimize perioperative risks. Cardiac catheterization was repeated and, if applicable, catheter interventions were performed in case that cyanosis aggravated during follow up or signs of cardiac decompensation due to volume overload were detected. Patients were electively scheduled for surgery with the goal of establishment of antegrade pulmonary blood flow and unifocalisation of big MAPCAs without double supply.

Children with adequate native pulmonary artery size were treated by VSD closure and either transanular patch or RV-PA conduit implantation. If available, an aortic or pulmonary homograft was utilized to reconstruct the right ventricular outflow tract. Apart from that, bovine jugular veins, a few autologous pericardial valved conduits as described by Schlichter et al. (11) and decellularized xenogenous conduits were used during a limited period of time as RV-PA conduits. The decision for single stage correction was made finally intraoperatively depending on actually achieved or present diameter of the pulmonary vascular bed. A later described flow study can contribute to ease arbitration. Pulmonary artery pressure was routinely measured intraoperatively after VSD closure by a pulmonary artery line.

Patients with hypoplastic native pulmonary arteries and MAPCAs without double supply were treated by unifocalisation and RV-PA conduit implantation as first surgery to avoid postoperative suprasytemic right ventricular pressure. During presurgical catheterization, MAPCAs with double supply were coiled, if this was tolerated well by the patient. VSD closure was performed later after satisfying growth of the pulmonary vascular bed.

Only in a small number of patients with diminutive vessels, in whom conduit implantation did not seem to be feasible, the first surgery was an implantation of a modified Blalock Taussig shunt or a central shunt in order to stimulate growth of the native pulmonary arteries. Surgery was also performed in cases of preoperatively severe life threatening cyanosis as a matter of last option. Recurrent stenoses of the pulmonary arteries were treated interventionally by balloon dilatation, stent implantation or surgically by a patch plasty.

In staged repair cases the decision for VSD closure was made after cardiac catheterization and pulmonary resistance assessment. The Nakata index, the Qp/Qs ratio, saturation and echocardiographic shunt direction were used as indicators. A Nakata index of at least 120 mm²/m² and arterial saturation of 80%–85% is regarded as indispensable for a successful VSD closure. This implicates a Qp/Qs ratio > 0. Recently also a standardized intraoperative pressure measurement protocol of the pulmonary arteries was introduced in order to optimize this decision making. Therefore, pulmonary artery reconstruction and the distal RV-PA conduit anastomosis was completed. Then patients were intraoperatively ventilated and the arterial cannula was switched from the aorta to the RV-PA conduit. This right heart bypass flow was increased up to 3 L/min/m² and pulmonary artery pressure was measured invasively. A VSD closure was performed if mean pulmonary artery pressure did not exceed 25 mmHg using full flow on isolated right heart bypass. A four millimeter shunt at the atrial level was left open. This measurement technique was used in 8 procedures in 6 patients and led to a VSD closure in 5 out of 8 procedures.

The included patients were followed from birth or first observation at our center until last observation follow up or death. Patients lost to follow up were included until last follow up.

The cohort was divided into two subgroups. Subgroup “PDA” contains all patients with pulmonary atresia and ductus dependent pulmonary circulation without MAPCAs (29 pts.; 38%). Subgroup “MAPCA” merges all patients with MAPCA dependent pulmonary perfusion (47 pts.; 62%). The maximal number of MAPCAs was 7, a median of 3 MAPCAs per patient was calculated. The majority of MAPCAs originated from the descending aorta, but also from the aortic arch, the normal or aberrant subclavian artery, the brachiocephalic trunk, the right coronary artery (1 pt.), the mammary artery (1 pt.) and the abdominal aorta (1 pt.).

Pulmonary arteries were non confluent in 34% of this subgroup. No central pulmonary arteries at all could be found in 11 patients (23% of the subgroup). Eight patients with a near total pulmonary stenosis and minimal antegrade flow, but dominant MAPCAs were also included in this group for the reason of equal surgical management.

Follow-up controls usually involved annual physical examinations and echocardiography. In those with diminutive native pulmonary arteries, cardiac catheterizations and interventions such as balloon dilatation and stenting of the pulmonary arteries were performed. If patients presented with severe pulmonary regurgitation after transanular patch usage, cardio magnetic imaging was done to assess right ventricular function and volume. In case of right ventricular dysfunction due to pulmonary and tricuspid regurgitation or significant right ventricular dilatation, an implantation of a valved RV-PA conduit was scheduled.

Data were collected retrospectively from patients' medical records and statistically analyzed. Data were compared between the two subgroups.

## Data availability statement

All relevant data are within the manuscript and its supporting information files.

## Ethics Statement

The institutional Ethics Committee (Ethics Committee of the Medical Faculty of the Johannes Kepler University) has approved the study under the no.1031/2020). Written patients informed consent was waived by the Ethics committee due to the retrospective character of the investigation.

## Statistics

All data of continuous variables were checked for normal distribution (test of normality: Kolmogorov-Smirnov with Lilliefors significance correction, type I error = 10%). Variables with normally distributed data were compared by the *t*-test for independent samples (test for variance homogeneity: Levene test, type I error = 5%); otherwise the exact Mann-Whitney *U* test was used. Dichotomous variables were compared by the Fisher's exact test, the other categorical variables by the exact chi-square test. For the comparisons of time to event variables, depicted by Kaplan Meier plots, the log-rank test was used.

The influence of presence of a patent ductus arteriosus, presence of MAPCAs, age at first surgery, age at VSD closure, sex and single stage full correction on the occurrence of exitus and on the occurrence of intervention (surgery or cath) after VSD closure was investigated by Cox regression analyses (stepwise forward based on the likelihood ratio approach).

The influence of sort of first RVPA-conduit on the occurrence of conduit replacement as well as the influence of type of first surgery on the occurrence of exitus and on the occurrence of intervention (surgery or catheter intervention) after full correction was investigated by univariate Cox regression analyses.

Furthermore, the influence of presence of a PDA, cardiopulmonary bypass time of first surgery, aortic cross-clamp time of first surgery, age at first surgery, sex and type of first surgery on 30 days mortality after first surgery and the influence of presence of a PDA, number of MAPCAs, age at first surgery, sex and presence of central pulmonary arteries on full correction achieved (=VSD closure achieved) was investigated by logistic regression analyses (stepwise forward based on the likelihood ratio approach).

The type I error was not adjusted for multiple testing. Therefore, the results of inferential statistics are descriptive only. Statistical analysis was performed using the open-source R statistical software package, version 3.6.1 (The R Foundation for Statistical Computing, Vienna, Austria).

## Results

### Operative data total cohort

The first surgeries took place at a median age of 47 days and the operative age ranged from 3 days to 17,5 years. 33 patients were surgically treated as neonates. Median weight at first operation was 3,9 kg (range 1,9–62 kg).

31 children (41%) could undergo single stage full correction consisting of VSD closure and either transanular patch plasty (15 pts.) or RVPAC implantation (16 pts.) and, if necessary, unifocalisation of important MAPCAs (5 pts.) at a median age of 12 days (range 3 days -13 years). Median bypass time was 173 min. (range 58–313 min.) and median aortic crossclamp time was 52 min. (range 36–146 min.) in this group. After single stage full correction, the children spent median 8 days (range 2–28 days) on ICU and 14 days (range 8–35 days) in hospital. 30 days mortality was 6%, but we counted four in hospital deaths after full correction (13%), one due to lethal bleeding after rupture in the region of a transanular patch during a pulmonary hypertensive crisis on pod 1; one patient deceased due to an inborn respiratory chain defect with untreatable hyperlactate acidosis on pod 3, one because of cerebral hypoxia after preoperative resuscitation at anaesthetization on pod 34, one of chronic respiratory failure due to cerebral impairment in CHARGE syndrome with microcephaly on pod 39. No late deaths occurred in this group.

The remaining 45 patients (59%) were not amenable to primary VSD closure due to a hypoplastic pulmonary vascular bed and were operated for the first time at a median age of 89 days (range 5 days - 17 years). 26 children primarily received a unifocalization of MAPCAs plus an RVPAC implantation without VSD closure, 16 pts. an RVPAC implantation only and 3 patients with diminutive pulmonary arteries a primary shunt procedure (2 modified Blalock Taussig shunt, 1 central shunt). In hospital mortality in this group was 18% (8 pts.) with a variety of causes for death: 4 pts. died due to hypoxia and cardiac failure, 2 due to embolism (1 pulmonary, 1 arterial on ECMO), one due to a severe combined immunodeficiency and sepsis and one because of lethal bleeding during a catheter intervention. 2 more patients died late during follow up after 159 days and 3,05 years from initial surgery due to bleeding in the cath lab and one due to hemoptysis after a reoperation. In 29 of these 45 patients (64,4%), the VSD closure could be performed after a median interval of 178 days (range 5 days—4,3 years) from initial surgery, three of them received a valved patch. The remaining 16 (21,1%) patients are either still awaiting ventricular septal defect closure (2 pts.), died before VSD closure (9 pts.), do not seem fit to tolerate the surgery (3 pts.) or were lost to follow up after the first surgeries (2 pts.).

In total 34 patients received a unifocalisation of MAPCAs. Altogether 78 MAPCAs were anastomosed to either the native pulmonary arteries or an RV-PA conduit, ranging between 1 and a maximum of 7 MAPCAs. The median number of unifocalised MAPCAs was 2.

### Long-term follow-up

The follow-up time of surviving patients was median 6,2 years after the first surgery with a maximum of 16,5 years. In this period the 14 previously described patients died due to hemorrhage (4), hypoxia (3), embolism (2) cerebral ischemia after CPR (2) and 3 due to non-cardiac malformations after an interval of median 26 days after the first operation. The estimated 10-year-survival rate after the first surgery is 80,5% ± 4,7% for the whole cohort of patients. A multivariate Cox regression analysis investigating the following covariates (PDA, presence of MAPCAs, age at first surgery, age at VSD closure, sex, single stage full correction) could not show a significant influence on the occurrence of exitus in our cohort. A logistic regression model analyzed the following factors regarding their influence on 30 days mortality (PDA, bypass time, aortic cross clamp time, age, sex, type of surgery), but also could not identify any significance (*p* > 0,05).

62 patients are alive and 74% could be categorized at their last follow up to be in clinical NYHA class I, 21% in class II and 5% in class III. The seven patients with an open VSD upon last follow-up, showed a mean peripheral oxygen saturation of 87 ± 8%, ranging from 73% to 95%.

24 patients (40%) underwent at least one reoperation after VSD closure. An estimated median surgery-free interval after ventricular septal defect closure of 7,5 years [95% CI:1,5–13,5 years] was calculated. Reoperations mainly concerned the right ventricular outflow tract addressing stenoses of the RVPAC and the pulmonary arteries. A univariate Cox regression demonstrated that in our cohort patients with aortic homografts had an earlier risk of reoperation in comparison to bovine jugular vein grafts (*p* = 0,039) or other sorts of RVPA connection (*p* = 0,001). The mean conduit diameters at first implantation were 10 ± 4 mm for the aortic homograft, 13 ± 2 mm for the bovine jugular vein, 9 ± 2 mm for the pulmonary homograft and 11 ± 1 mm in the group of other conduits. The median interval until conduit replacement was 4,8 ± 1,4 years.

193 catheter interventions were performed in the investigated period with a median of two interventions per patient (range 0–11). 100 stents were implanted in 35 patients with a maximum of 7 stents in one patient and 42 coils were used in 21 patients (range 0–5) in order to close MAPCAs or minor collaterals. 34 patients (56,7%) had at least one and up to 7 transcatheter interventions following VSD closure. The median estimated catheter-intervention-free interval after VSD closure was 1,8 ± 0,8 years. After VSD closure, an average of 2 interventions in 5 years were performed per patient. A multivariate Cox regression analysis examined possible variables for the occurrence of surgical or catheter interventions after VSD closure. Older age at first surgery (*p* = 0,015), initial presence of a PDA (*p* = 0,024) and single stage full correction (*p* = 0,009) were found as significant influence factors regarding reinterventions. A univariate model could not find an influence of type of first surgery on the occurrence of reintervention after VSD closure.

Subgroup comparison between Group PDA and Group MAPCA (see also Table 1).

**Table 1:** Subgroup comparison.

	Group PDA	Group MAPCA	*p*
*N*	29	47	
Median age at first surgery (days)	12	112	<0,001
Single stage full correction	83%	15%	<0,001
30 days mortality after first surgery	10%	10%	>0,999
VSD closure achieved	93%	70%	0,021
Median age at VSD closure (days)	11	457	<0,001
Median follow up after first surgery (years)	9,6	3,1	0,006
Total mortality	17%	19%	>0,999
Estimated 10-year-survival	81,9% ± 7,3%	79,1% ± 6,3%	0,881
Surgery after VSDC	44%	36%	0,601
Cath. intervention after VSDC	56%	58%	>0,999
Median time to either intervention after VSDC (days)	375	235	0,262
Median surgery-free interval after VSDC (years)	6,0	7,7	0,772

**Figure 1:**
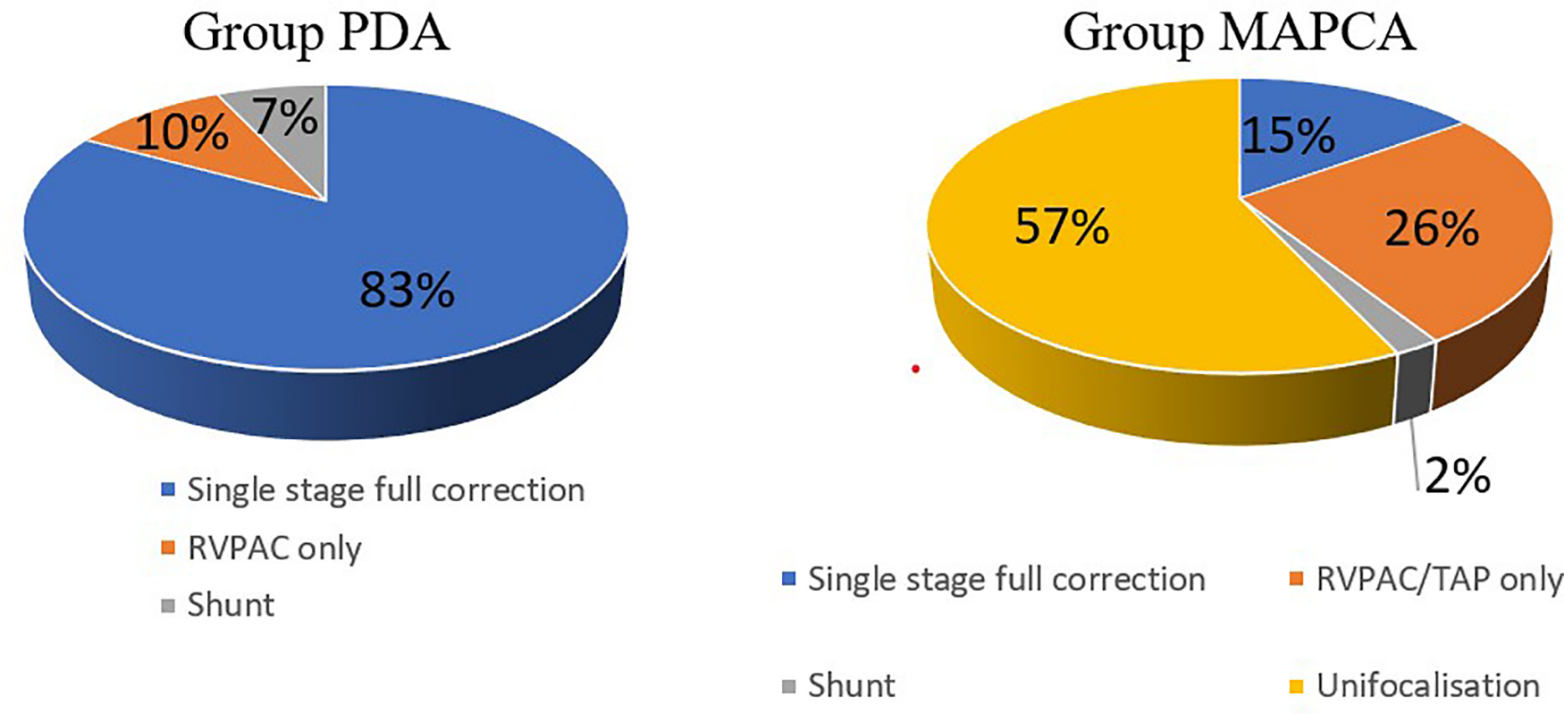
Primary surgical procedures (subgroup comparison).

The Group PDA consisted of 29 patients of whom 55% were female. Group MAPCA included 47 kids of whom only 43% were female. The following significant differences in surgical strategy between the two subgroups can be described (see Figure 1): In Group PDA single stage full correction was possible in 83% vs. 15% in Group MAPCA (*p* < 0,001) and the first operation was performed significantly earlier in Group PDA (median age 12 days vs. median 112 days in Group MAPCA) (*p* < 0,001). After their first surgery, patients of Group MAPCA stayed median 22 days in hospital compared to 17 days in Group PDA (*p* = 0,378). 14 children of Group MAPCA (30%) had an episode of ECMO during follow up compared to only 1 (3%) of Group PDA (*p* = 0,006).

**Figure 2:**
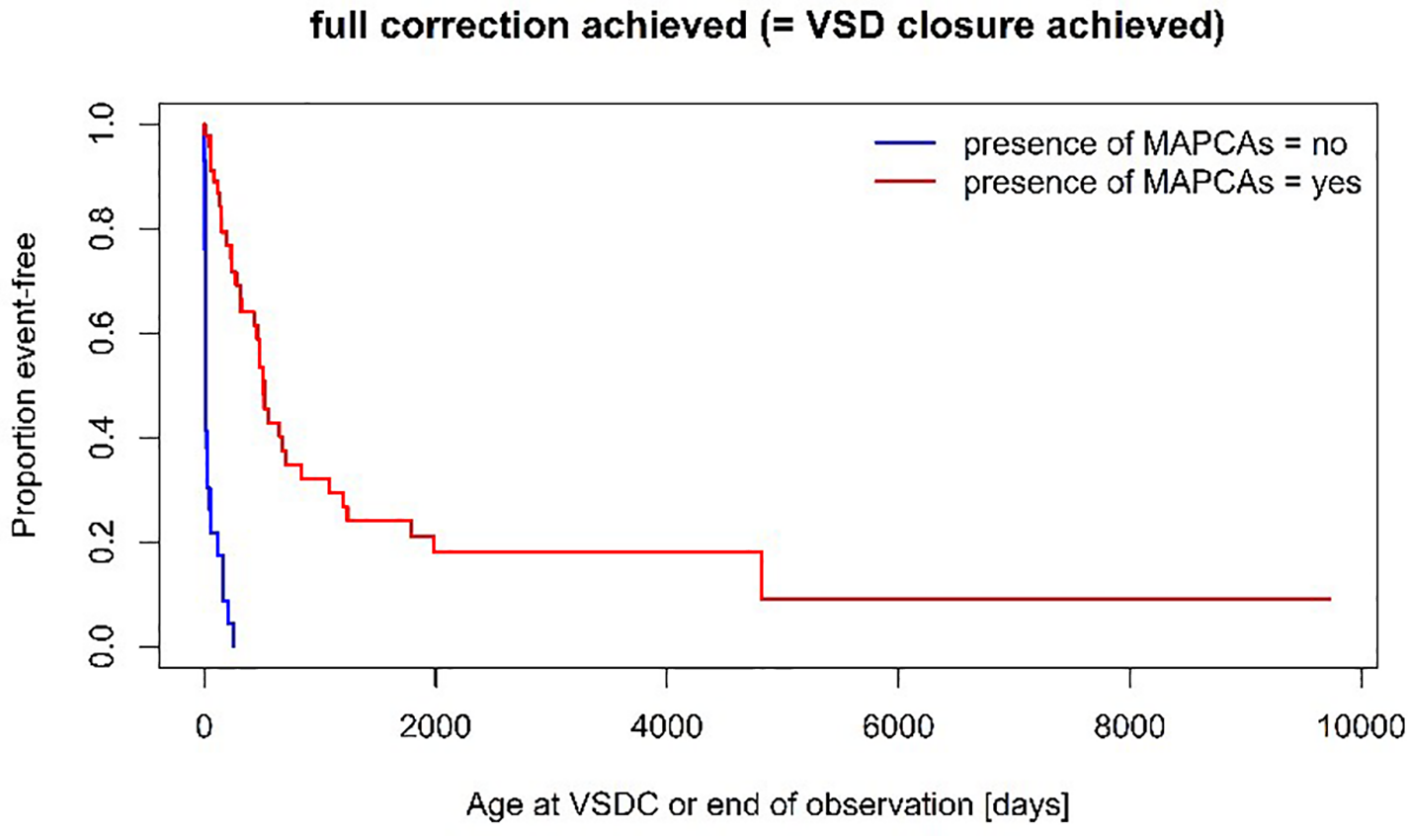
Time to VSD closure (log-rank test: *p* < 0,001).

A unifocalization was performed in 72% of the patients of group MAPCA (see Figure 1). A VSD closure could be achieved in 93% (Group PDA) vs. 70% (Group MAPCA) (*p* = 0,021) during the follow up period and it was achieved significantly earlier, at a median age of 11 days in Group PDA vs. an age of 457 days in Group MAPCA (*p* < 0,001) (see Figure 2). The median interval between first surgery and VSD closure in group MAPCA was 121 days (range 0–4,3 years). A logistic regression model analyzed possible factors regarding the likelihood of VSD closure (PDA, number of MAPCAs, age, sex, presence of central pulmonary arteries), but could not identify an independent one.

**Figure 3:**
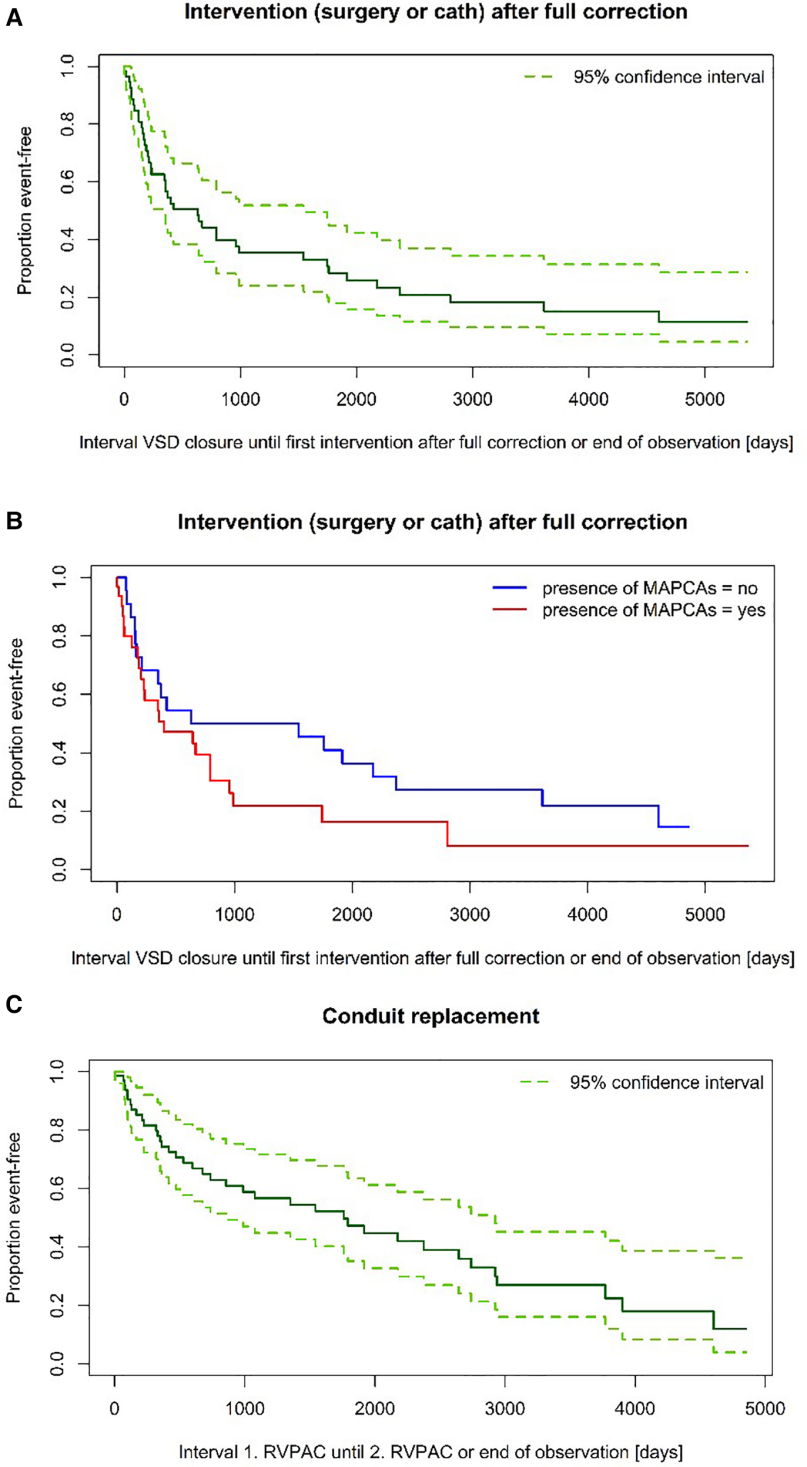
(**A**) Time to intervention after VSD closure. (**B**) Time to intervention after VSD closure—subgroup comparison (log-rank test: *p* = 0,149). (**C**) Time to RVPAC replacement.

A significant difference in surgery-free interval after VSD closure for patients with and without MAPCAs could also not be found (6 vs. 7,7 years) (*p* = 0,772). Neither was there a significant difference in catheter-intervention-free interval between the two groups (*p* = 0,432) (see also Figures 3A–C). Although the median follow-up period of Group MAPCA was shorter, more catheter interventions took place in these patients. 245 dilatations were performed in 43 patients (maximum: 27 dilatations in one patient) with an average of 4,5 dilatations in patients with and 1,1 dilatations in patients without MAPCAs. The mean number of stents per patient was 1,6 in children with and 0,8 in children without MAPCAs.

**Figure 4:**
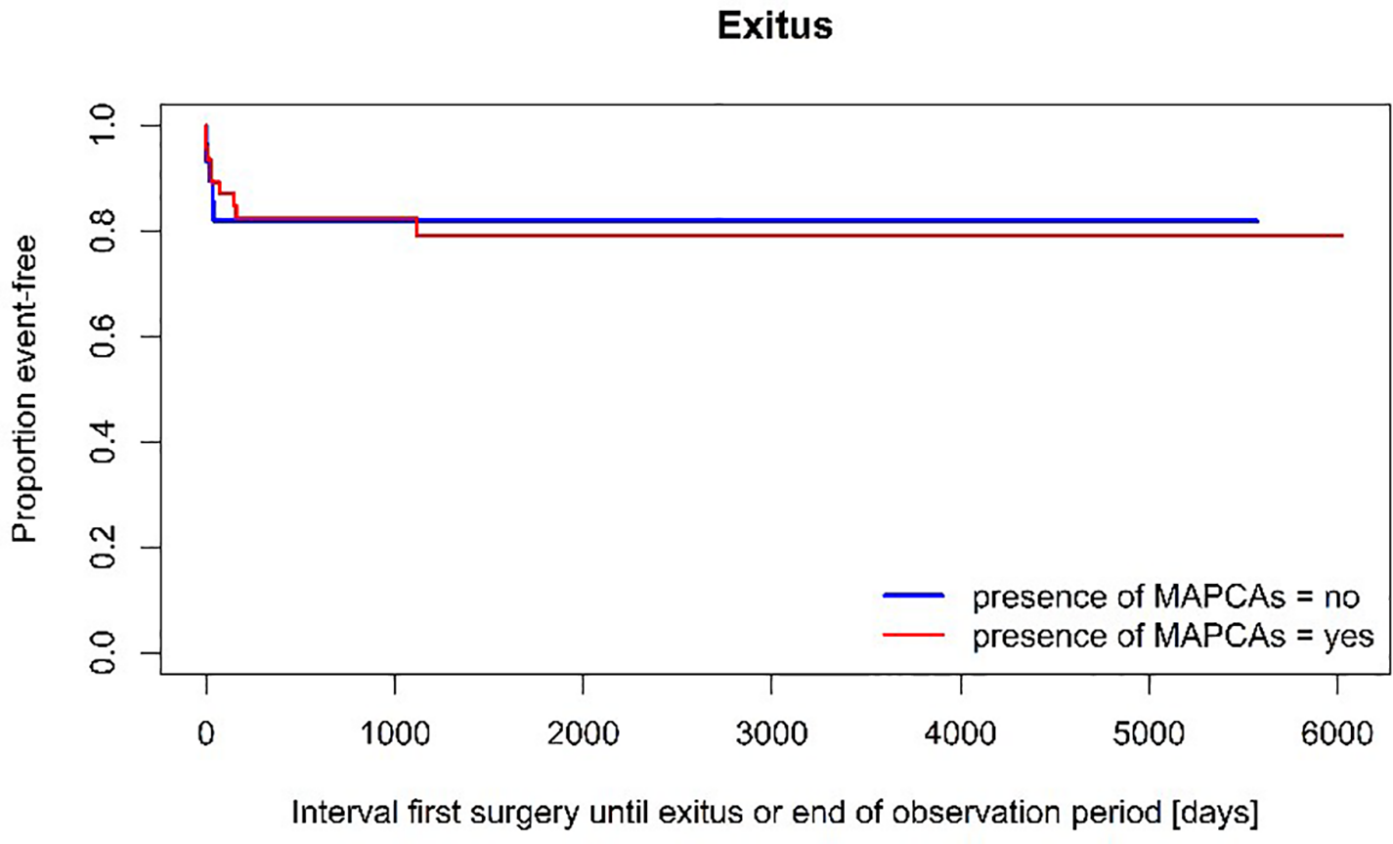
Survival—subgroup comparison (log-rank test: *p* = 0,881).

Overall mortality rate was comparable between the two groups. The 10-year-survival expectancy in patients with MAPCAs was estimated at 79,1% ± 6,3% compared to an estimated 81,9% ± 7,3% in the group with PDA (*p* = 0,881) (see Figure 4).

## Discussion

**Figure 5:**
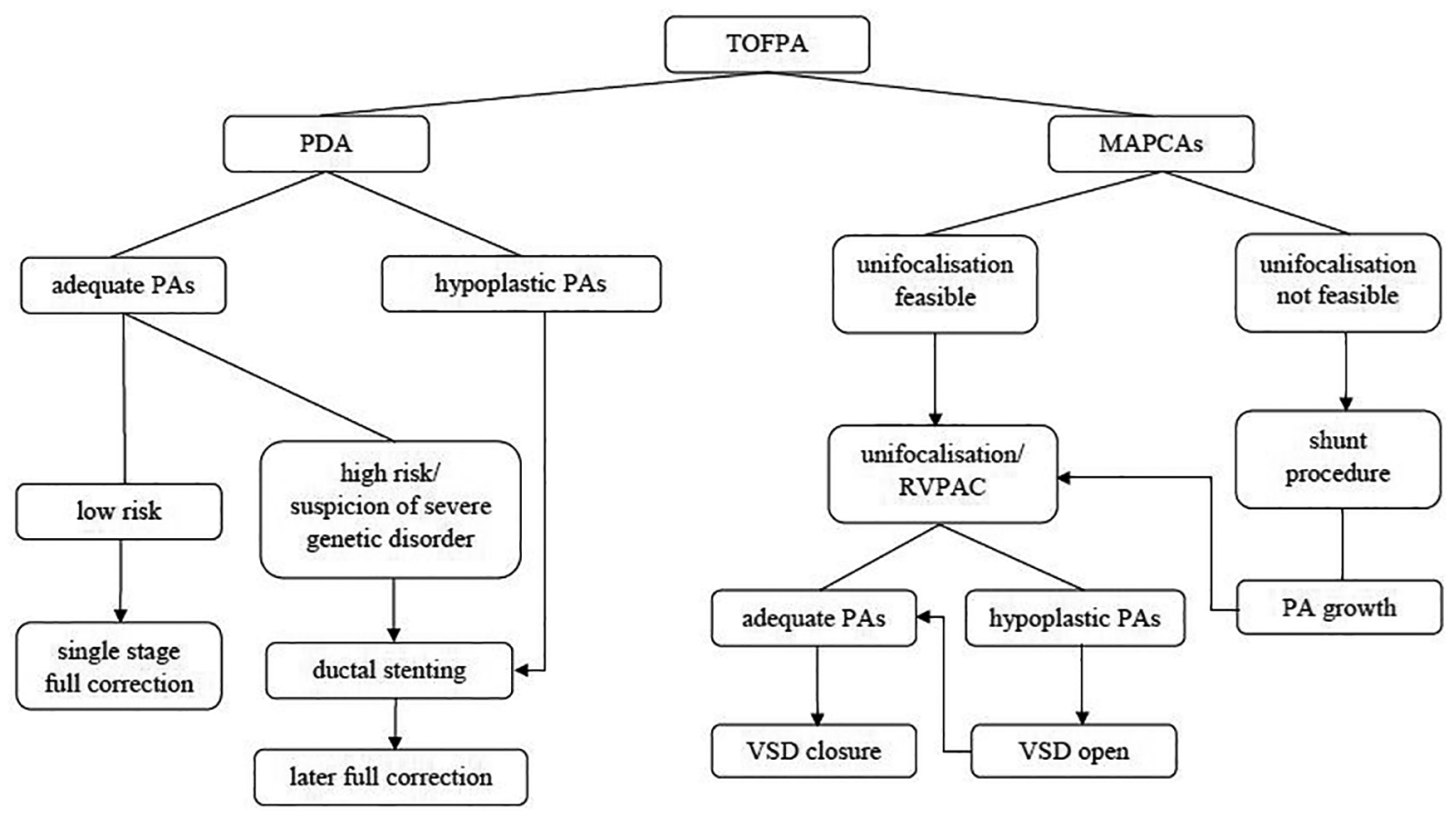
Decision tree.

This study is meant to give an overview of procedures performed in the heterogenous cohort of all consecutive unselected patients with TOFPA reaching a single middle volume center comprising patients with very diverse anatomy. Our approach was to attempt complete repair as early as possible in order to preserve right ventricular function and cease shunting. At our center we predominantly pursued a unifocalization strategy in patients with MAPCAs without double supply. In this group a VSD closure could be achieved in 70% during a follow up period of 3,1 years, which was unsurprisingly lower than in children with initially open ductus arteriosus (VSD closure rate 93%). Nevertheless, a single stage full correction was possible in only 83% of PDA patients and hypoplasia of the pulmonary arteries can also be present in this allegedly simpler lesion. Ductal stenting should be considered as an alternative in high risk patients to postpone on pump surgery to a safer time slot and to allow for a more thorough evaluation in those patients considered high-risk due to non-cardiac defects (see Figure 5). Nevertheless, the resulting shunt physiology does also imply certain risks.

Restenoses at the level of the RVPAC and adjacent regions are commonly seen during follow up and are reason for surgical and catheter interventions in both groups (12, 13). The direct comparison of interventions in our series showed a higher mean number of interventions in MAPCA patients, but after VSD closure, time to intervention is comparable between the two groups and median only 1,8 ± 0,8 years. The occurrence of interventions after VSD closure is more likely in older patients (many of them in group MAPCA) and less likely in children with single stage full correction. The smaller aortic homografts used in the neonatal period led to earlier conduit reoperations than with the bovine jugular vein conduits used more deliberately at an older age. Nevertheless, the surgery free interval after VSD closure was satisfying with median 7,5 years.

The mortality rate in patients with single stage full correction was lower than in children with staged approach (13% vs. 21%), but higher than previously expected. Two of the 4 deaths of this group were caused by non-cardiac malformations or metabolism disorders preoperatively not diagnosed to full extent. In total 21% of mortality cases in our series can be attributed to non-cardio-pulmonary genetic conditions. The study center did also offer surgical therapy in extreme cases with bad preoperative conditions (e.g., severe preoperative hypoxemia due to extremely unfavorable anatomy) as a matter of last option which led to losses because of intolerable cyanosis.

In the last six years of the investigated period there was only one case of in hospital mortality out of 24 patients. Hence, growing experience with the treatment of this challenging anatomy as well as ECMO possibility (available at the center only since 2007) may be of decisive influence on success, as 43% of deaths happened before ECMO therapy could be offered.

Tetralogy of Fallot with pulmonary atresia is a challenging malformation because of hypoplasia of the pulmonary vascular bed. Bertranou et al. depicted an estimated 10-year-survival of 8% for these patients without surgery (14). Nowadays most patients are surgically treated in infancy, but successful treatment after late presentation has also been described (15).

Several approaches have been made to stimulate pulmonary artery growth with the aim to achieve a pulmonary vascular resistance low enough for closure of the ventricular septal defect without right ventricular pressure overload. As a first surgical step, unifocalization and antegrade connection of the native pulmonary artery bed is proclaimed by many authors to be essential for pulmonary artery development (2–4). A review of the most recent investigations reported this strategy to be probably the most commonly used one internationally (16). On the other hand, some centers, especially the Melbourne group, are successful in using primary shunt procedures to increase pulmonary flow and abstaining from integrating MAPCAs into the pulmonary tree because of the long-term experience of severe stenoses and thromboses of MAPCAs (17). At date a superiority of one approach is definitely not clear as both strategies can be very successful. Most studies achieved complete repair rates between 60%–80% in TOFPA cases with MAPCAs with a maximum of a 88% VSD closure rate in the largest series reported by the Stanford group (2, 3, 6, 12, 16). The unifocalization technique was also applied successfully in the extreme cohort of patients with absent central pulmonary arteries with a comparable VSD closure rate of 80% (18).

Despite the significant mortality rate early after initiation of treatment, the long-term survival expectancy was good showing only one case of death three years after the first surgery in a patient with severely impaired pulmonary vasculature. The estimated 10-year survival rate of approximately 80% does not show significant differences between the two groups (*p* = 0,881), surprisingly in face of the heterogenous anatomic complexity. This may be owed to the small number of investigated patients but fits in the range of overall mortality revealed by the latest review (78%–85% long-term survival) (2, 13, 16).

The need for intense treatment during follow up in both groups is reflected in the high number of interventions after complete repair (19). The fact that there was no lethal event in context with a reoperation after VSD closure is promising. The majority of surviving patients showed a good quality of life at their last follow up with 95% being in NYHA class I or II. But the high rate of genetic disorders is a special interdisciplinary challenge in this complex malformation (20) and has been reported to be of negative influence on survival rates (7).

## Limitations

The heterogenous anatomy and the small overall number of patients with this rare lesion compared to specialized centers make conclusions difficult. Therefore, the character of the study is merely descriptive. The long investigational period merges patients with different treatment facilities and experience although all of them were operated at a single center. Besides, some patients from abroad could not be followed as national citizens.

## Data Availability

The raw data supporting the conclusions of this article will be made available by the authors, without undue reservation.
